# Research on the impact of generative AI interaction modes on the improvement of school-age children’s early reading ability and college students’ auxiliary intervention

**DOI:** 10.3389/fpubh.2026.1926167

**Published:** 2026-09-23

**Authors:** Yanjiao Dong, Guoyan Li

**Affiliations:** 1 Anyang Normal University, Henan, China; 2 Shinawatra University, Pathum Thani, Thailand

**Keywords:** college students, early reading ability, early reading for school-age children, generative artificial intelligence, interaction modes

## Abstract

**Background:**

Against the backdrop of digital empowerment, generative artificial intelligence has been widely applied in school-age children’s reading education, and digital reading has become one of the primary types of environmental exposure for school-age children. However, school-age children face challenges such as insufficient age-appropriate content and uncontrollable physical and mental strain. There is an urgent demand for a public health intervention model that balances reading-related health benefits, prevents and controls digital exposure risks, avoids physical and mental health impairments, and adapts to school-age children from diverse socioeconomic backgrounds.

**Methods:**

A retrospective control study design was used in this study. A total of 100 cases of school-age children aged 6–12 were collected from the standardized intervention archive of our institution from September 2025 to December 2025. According to the intervention plan actually accepted in the archive records, they were divided into the collaborative intervention group and the AI-only reading group with 50 cases in each group. In the AI-only reading group, the recorded intervention is purely generative AI independent reading intervention (college students are only responsible for equipment operation and maintenance, and do not participate in reading guidance and emotional intervention), while in the collaborative intervention group, the recorded intervention is the structured auxiliary collaborative intervention of the whole process of generative AI voice multi-round interactive reading combined with standardized training for college students. The intervention period is 12 weeks, three times a week, and each time is 20–25 min. Retrospective analysis of longitudinal data at baseline, 6 weeks of intervention, 12 weeks of follow-up and 4 weeks of follow-up, with the comprehensive score of early reading as the only preset main outcome index, repeated measurement analysis of variance, independent sample *t*-test and chi-square test were used to analyze the inter-group and time effects, Cohen’s *d* was used to evaluate the effect, and the test level was *α* = 0.05.

**Results:**

Retrospective archive data shows that, after intervention, the collaborative intervention group achieved significantly higher composite early reading scores, phonological awareness, vocabulary comprehension accuracy, reading attention duration and reading interest scores than the AI-only reading group (*p* < 0.05). The main effects of group, time and the group-time interaction were all highly statistically significant (*p* < 0.01), with an effect size of Cohen’s *d* = 0.50 representing a moderate intervention effect. The collaborative intervention group also showed significantly higher intervention adherence rate, AI interaction completion rate, reading task completion rate and frequency of active participation than the AI-only reading group (*p* < 0.05). In terms of digital health-related outcomes, the collaborative intervention group had significantly lower incidence of adverse psychological states, incidence of prodromal signs of visual fatigue and digital eye strain symptoms, and study dropout rate (*p* < 0.05). Among the digital health-related safety indicators, the incidence of negative emotions, visual fatigue and research dropout rate were significantly lower than those of the AI-only reading group (*p* < 0.05). The archived records showed that the whole screen duration was strictly in compliance, and there was no privacy leakage and serious adverse events. The 10% lost visit rate was controllable, and the complete rate of informed consent and the qualified rate of implementation were all 100%, and there was no privacy leakage and serious adverse events.

**Conclusion:**

The generative AI interaction mode paired with college student auxiliary support is associated with sustained improvement school-age children’s core early reading ability via cumulative short-term follow-up effects. Meanwhile, this collaborative model enables effective management of digital environmental exposure risks and may reduce risks related to visual and psychological health among school-age children. The intergroup differences remained stable with reliable effect sizes. This intervention model can be may provide a reference for future implementation as a digital public health intervention targeting school-age children’s early reading, and provides preliminary empirical evidence for the standardized application of AI-supported education for school-age children. This retrospective study only reflects the differences between groups of compound intervention programs in archived cases, and cannot establish strong causal inference.

## 1 Introduction

6–12 years old is the golden window of school-age children’s reading ability development. The developmental levels of school-age children’s reading interest, phonological awareness, vocabulary decoding, text comprehension and reading attention duration lay the groundwork for their subsequent academic performance and lifelong learning capabilities, rendering this field a core public health intervention target among school-age children (1). Traditional reading activities for school-age children mainly depend on paper picture books, parental company and teacher guidance. Although such approaches feature inherent strengths including immersive reading experience and low digital exposure, they are plagued by practical public health drawbacks: fixed reading modes, inadequate personalized health guidance, unequal allocation of professional health accompaniment resources between urban and rural regions, and insufficient health equity. Consequently, these traditional methods fail to satisfy differentiated reading and health development demands of vulnerable groups, such as school-age children from low-income and rural households (2). Driven by iterative upgrades of large language model (LLM) technology, generative artificial intelligence (AI)—endowed with merits including natural language dialogue, contextualized story narration, real-time Q&A, personalized difficulty adaptation and positive emotional reinforcement—has rapidly permeated household and school-age children’s educational scenarios. As a novel supporting medium for school-age children’s digital reading, it delivers innovative technical backing for public health interventions targeting school-age children’s reading (3).

The application of generative AI in school-age children’s reading education is currently undergoing explosive expansion; however, when viewed from the perspectives of public health governance for school-age children and population health protection, existing applications carry multiple non-negligible health risks and governance deficiencies. Most commercial AI reading products lack filtering mechanisms for age-appropriate content tailored to school-age children, and their algorithmic recommendations tend to be entertainment-focused and fragmented. This easily causes school-age children to lose concentration and engage largely in superficial reading, hindering the cultivation of in-depth reading comprehension abilities (4). Current generative AI-assisted reading intervention primarily prioritize technical function demonstration while ignoring the developmental rules of school-age children’s physical and mental health, including eye health, mental strain, and emotional perception. Long-duration screen exposure may trigger secondary problems such as visual fatigue, negative emotional agitation, and electronic device addiction tendencies (5). More importantly, health inequity gaps remain prominent. Rural regions and low-income households have no access to professional staff to conduct standardized health supervision over digital reading, leaving vulnerable school-age children exposed to high-risk AI reading environments without standardized interventions. Accordingly, the disparity in digital reading health between urban and rural school-age children, as well as children from disparate socioeconomic backgrounds, keeps widening, the imbalance of digital health fairness is prominent (6).

The existing related research focuses on the single effect verification of generative artificial intelligence on children’s reading performance, emphasizing on technical efficiency evaluation, lacking systematic research in the dimensions of electronic environment exposure risk prevention, children’s physical and mental health damage prevention, group inclusive public health intervention, health equity, etc., and separating the educational reading outcome from public health outcomes such as digital exposure and psychological and visual health. Meanwhile, few existing studies involve trained college student volunteers receiving standardized public health training in collaborative interventions, failing to explore the core value of human companionship in protecting school-age children’s digital health (7). College students have sufficient spare time, proficient communication skills and a strong sense of responsibility. After standardized reading guidance training, they can effectively undertake multiple tasks: monitoring eye-use behaviors, maintaining reading order, offering emotional counseling, guiding AI interaction procedures, and recording process data. Such volunteers can make up for the defects of pure machine interaction—insufficient humanistic care and emotional companionship—thus realizing complementary advantages between generative AI technological empowerment and human-centered auxiliary interventions (8).

Nevertheless, academia has not yet established a unified standardized system to calibrate AI reading environmental exposure intensity, public health intervention frequency and matching human health companionship standards. Current literature lacks multi-time-point longitudinal follow-up controlled data, as well as integrated evidence-based quantitative evidence incorporating statistical effect sizes, physical and mental digital health-related safety indicators and population health equity, together with group-time interaction effects (9, 10). Therefore, it is urgent to construct a safe, controllable, age-adaptable and quantitatively effective generative AI-assisted reading intervention, and integrate college student auxiliary support to build a synergistic collaborative mechanism. Adopting a retrospective observational research design, this study systematically verifies the improvement effects of this human–machine synergistic intervention model on school-age children’s comprehensive early reading ability, phonological-semantic cognition, visual health, psychological-emotional status and healthy reading behaviors, and quantifies its preventive and controlling efficacy against screen exposure and adverse physical and mental outcomes.

This study holds that children’s digital reading is a typical crowd health intervention scene, which has complete public health research attributes. The exposure of digital screen at the group level belongs to group environmental risk factors, myopia and emotional behavior problems cause high public health damage to children, and the differences in family economy and educational resources at the fair level bring about healthy differentiation of digital reading, which belongs to the category of health equity research. At the intervention level, standardized human-computer collaborative intervention can reduce the health risks caused by electronic exposure and improve children’s cognitive health level, which belongs to the category of crowd health promotion intervention. At the ethical governance level, the protection of minors’ data privacy, the control of screen length and the exit mechanism without punishment are the core contents of children’s public health ethics, so it has sufficient theoretical logic to analyze the AI reading intervention of school-age children under the framework of public health. Based on the retrospective analysis of the cases filed by the complete intervention of our institution, this study presupposes the following hypothesis of retrospective verification, that is, the collaborative intervention group with standardized college students’ structured auxiliary intervention has higher comprehensive scores, phonological awareness, vocabulary understanding, concentration duration and average reading interest in early reading than the AI-only reading group with AI-only reading intervention. The intervention compliance rate, AI interaction completion rate, reading task completion rate and children’s active participation frequency in the collaborative intervention group were better than those in the AI-only reading group. The incidence of adverse health events related to digital exposure in the collaborative intervention group was lower than that in the AI-only reading group, and there was an interactive effect between the intervention time and the intervention mode. With the extension of the intervention period, the difference in reading ability scores between the collaborative intervention group and the AI-only reading group continued to expand, and the follow-up related indicators did not drop significantly.

This study is a retrospective control study, and the data comes from the archived cases that have completed standardized intervention in our institution, including intervention execution records, four-point standardized evaluation data, four-week follow-up records, volunteer training quality control accounts and informed consent archiving. The study did not carry out pre-intervention allocation and prospective intervention manipulation, but only carried out retrospective grouping analysis according to the intervention plan actually accepted by the cases, which is different from the prospective intervention trial that actively allocated subjects. Its core innovation is to build a retrospective control model of human-computer collaborative compound intervention based on real archived cases, distinguish between AI-only reading intervention and AI combined with structured assistance of college students, restore the intervention effect of real service scenarios, and build an integrated retrospective evaluation system that integrates educational reading outcomes and digital exposure physical and mental health safety outcomes, taking into account learning efficiency and children’s public health protection. The feasibility of low-cost Pratt & Whitney man–machine collaborative intervention was evaluated based on standardized training volunteer intervention account, and repeated measurement and analysis were carried out by using four-point longitudinal archive data to capture the dynamic change of intervention effect and the stability of short-term follow-up, so as to make up for the implementation deviation between prospective controlled research and real service scenarios.

## 2 Materials and methods

### 2.1 Study design

This study adopts a retrospective control study design. The data source is the complete archive case files of school-age children who have completed a complete set of standardized human–machine collaborative AI reading intervention in our institution from September 2025 to December 2025. The standardized intervention period is 12 weeks, and the short-term follow-up observation archive records are kept for 4 weeks after the intervention. All files are archived until December 31, 2025.

Archived standardized evaluation records at four points: baseline (before intervention), 6th week of intervention (mid-term), 12th week of intervention (terminal stage), 4th week after intervention (follow-up period), archive scale, on-site behavior observation record and safety and health indicators ledger.

The whole process of the original file intervention strictly follows the four principles of children’s public health protection: maximizing children’s health interests, voluntarily withdrawing at any time without punishment, anonymously desensitizing the privacy of health data, and minimizing the risk of electronic exposure; In this study, the questionnaires of complete family socio-economic status, family daily reading environment and other mixed variables in the archives were retrospectively extracted, all of which were included in the baseline balance test. Based on the archived baseline data, the selection bias, information bias and mixed bias were controlled to ensure that the baseline demographic, family environment, audio-visual development and initial reading ability of the two groups of archived cases were balanced and comparable.

### 2.2 Sample size estimation

In this retrospective study, the consecutive archived intervention cases in the same period were retrieved. Before the original intervention, the sample size was estimated based on the comprehensive score of early reading (the total score of ERAS scale), with a two-sided significance level of *α* = 0.05, a power of 1 − *β* = 0.90, and a preset between-group effect size of Cohen’s *d* = 0.50, PASS software was used to estimate the sample size for a parallel-group controlled design. The results showed that a baseline sample size of 22 participants was required for each group; taking into account a 10% loss-to-follow-up rate during the study, a minimum of 25 participants per group was required. Given that this study is an educational intervention study involving school-age children, and considering potential confounding factors such as fluctuations in intervention adherence, interference from the home environment, and a relatively long follow-up period, the total sample size was ultimately set at 100 participants—50 in the collaborative intervention group and 50 in the AI-only reading group—to further enhance the robustness of the statistical analysis.

### 2.3 Inclusion criteria

① Archived cases are school children aged 6–12; ② Normal intellectual development is defined as a score of ≥85 on the Wechsler Intelligence Scale for Children (WISC); normal visual and auditory development is defined as uncorrected or corrected visual acuity of ≥1.0 and a hearing screening threshold of <25 dB; participants must be able to independently cooperate with paper picture book reading and standardized scale assessments; ③ Guardians voluntarily sign a written informed consent form, committing to complete the entire 12-week intervention and 4-week post-intervention follow-up, and to cooperate with assessments at each time point; ④ No severe developmental delays, mental or behavioral abnormalities, or organic language disorders; able to participate normally in Generative AI voice interaction and paper picture book co-reading; ⑤ Daily digital exposure duration complies with school-age children health guidelines, with no signs of addiction to or excessive use of electronic devices.

### 2.4 Exclusion criteria

① Archive medical records to diagnose autism spectrum disorder, attention deficit hyperactivity disorder, severe language retardation, learning disabilities and other diseases that directly affect reading performance and effectiveness of assessment; ② Received professional systematic reading training, special literacy training or similar digital reading intervention in recent 3 months; ③ The guardian has poor compliance, cannot accompany school-age children to participate in the fixed intervention period on time, or is not suitable for screen reading due to serious eye diseases; ④ Those who participated in other similar school-age children’s education intervention projects at the same time, and were at risk of intervention contamination and result interference. ⑤ Those who continue to take antipsychotics, central stimulants, sedatives and other drugs that can interfere with cognitive function and attention in the past 3 months.

### 2.5 Rejection and dropout standards

① According to the file records, during the intervention period, they voluntarily applied to withdraw from the study, lost their follow-up for more than 2 times, or were excluded if the overall intervention compliance was less than 85%; ② Those who have sustained irritability and resistance, severe visual fatigue, dizziness and discomfort during the intervention, and cannot continue to complete the intervention process, will be treated as dropout cases; ③ The missing rate of key assessment indicators and behavior observation data is more than 20%, and the answers of the scale are invalid, which is not included in the final statistical analysis; ④ In the middle of the course, privately participate in other interventions such as off-campus reading tutoring and online literacy courses, and those who undermine the homogeneity of inter-group interventions will be eliminated.

### 2.6 Grouping method

This study is a retrospective control study. According to the actual intervention situation in the records, it is divided into AI-only reading group with 50 cases and collaborative intervention group with 50 cases. In this study, a single-blind scheme was implemented for the outcome evaluation, and the evaluators did not know the grouping information of the school-age children in the whole process, and the grouping confidential letter was unpacked after all the data statistics were completed. Before the intervention, the baseline data of school-age children’s age, gender composition, basic cognitive level and initial reading ability in the two groups were balanced, and there was no statistical difference between the two groups (*p* > 0.05), which was comparable. The two groups unified the frequency, period and duration of intervention, only the intervention modes were different, and the rest of the environment, reading materials and assessment tools remained completely consistent.

The original intervention outcome evaluation implemented a single blind scheme: the evaluators did not know the subjects’ grouping in the whole process, and the secret letter of grouping was filed with the file, and it was unpacked before this retrospective statistics; Based on the balance test results of all mixed indicators of the archived baseline, the archived baseline records of the two groups were *p* > 0.05, and the archived baseline data between the two groups were comparable. In the initial intervention stage, the frequency, period, duration of intervention, picture book materials, AI system and evaluation tools of the two groups were completely archived, and only the intensity and function of real-life assisted intervention were different between the two groups.

### 2.7 Intervention implementation plan

Both groups accepted the conventional reading mode of traditional paper picture books. Parents or class teachers will carry out regular reading and free questions and answers to maintain the natural regular reading state. The AI-only reading group completed the accompanying operation by trained college student volunteers with the same qualifications, eliminating the confounding interference caused by the qualifications and ages of the accompanying personnel. The unified intervention period of the two groups is 12 weeks, and the intervention is fixed three times a week. The duration of a single intervention is strictly controlled for 20–25 min, and the eye distance, sitting posture and digital exposure duration are regulated throughout the whole process, and the frequency and duration are not allowed to increase or decrease at will. The only difference between the two groups was recorded on file. In the AI-only reading group, college students only undertook equipment operation and maintenance and order maintenance, but did not participate in reading guidance, emotional counseling and real-time eye intervention. After a full set of standardized training and 1:1 structured auxiliary intervention, college students in the collaborative intervention group completed multiple functions of eye control, emotional counseling, reading guidance and behavior recording simultaneously, and all the operation processes were archived.

#### 2.7.1 Intervention scheme of the control group: pure generative AI interaction modes

The original intervention adopted V4.1, a large model for customized reading of Tongyi Lingma scientific research, with 216 books of graded picture books, and the grading standards were archived with reference to the Graded Reading Guide for School-age Children in China. The interaction logic is locked by the background fixed script, and the archive record of interaction parameters cannot be modified manually; Pure voice interaction, low brightness static graphics and text, no animation special effects; Built-in five modules: reading aloud, hierarchical questioning, vocabulary analysis, positive encouragement and logical guidance, with 3–5 rounds of interaction at a time, with a speech speed of 120 words/min, age-appropriate content filtering, no advertising, low blue light and complete privacy data encryption system. College student volunteers only perform basic operation archiving: adjusting tables and chairs at the beginning, turning on equipment, and handling power failure; AI independently completes all reading interactions, questions, vocabulary explanations and story summaries; Volunteers do not participate in reading questions and answers, do not take the initiative to guide thinking, do not correct bad eyes in real time, and do not take the initiative to channel negative emotions. Only at the end of the intervention, the basic participation data are recorded uniformly, and the dynamic health intervention in the process is not carried out.

2.7.2 The intervention scheme of the collaborative intervention group adopts the multi-round interaction of generative AI voice combined with the whole process of college students’ standardization to assist collaborative intervention. The college students’ volunteers with the same origin as the AI-only reading group have the same source of volunteers, training content and personnel qualifications, and only the operating norms of accompanying intervention are distinguished.

(1) AI interaction module: completely homologous to the AI-only reading group, with no difference in parameters, picture book library and interactive logic archive records, eliminating the interference of AI variables;
(2) The core archive content of structured auxiliary intervention for college students (supplement the intervention intensity and specific functions, and improve the abstract description): All college students volunteers complete the archive account of 24 h of pre-job standardized training in 3 days, and five modules: scientific research norms (6 h), children’s psychological counseling practice (6 h), eye health control (4 h), AI system operation guidance (4 h) and reading behavior observation records (4 h); Theory + practice double assessment archive, the pass rate of 96.3% can be employed; once a week, the intervention period will focus on supervising the re-filing records, and rectify the irregular operation in a unified way; the implementation of 1:1 full-time companion archive record for one college student corresponding to one child is different from the AI-only reading group’s only equipment operation and maintenance mode. The core intervention function archive records include: ① Dynamic control of electronic exposure risk: real-time correction of bad eye posture such as looking down, looking too close to the screen and sitting sideways, and controlling the duration of a single screen in the whole process; ② Psychological and emotional pre-intervention: identify negative emotions such as irritability, resistance, crying and avoidance in real time, and immediately carry out persuasion and appeasement to reduce reading conflict; ③ Reading participation guidance: cooperate with AI interactive nodes, moderately encourage children to actively follow, ask questions and repeat, and improve task participation; ④ Standardized process record: record the duration of single concentration, frequency of active interaction, visual discomfort and emotional expression in real time, and realize full-quantitative quality control archiving of the process.

### 2.8 Original intervention consistency guarantee and quality control filing system

In the original intervention stage, the quality control ledger was completely retained, and this review was synchronized for verification: ① Volunteer supervision mechanism: centralized supervision and re-filing records every week, random sampling of 20% of the whole intervention video archiving, verification of operational norms, and secondary retraining of unqualified volunteers; ② AI-generated content monitoring mechanism: all AI interactive texts and voice records are kept in the background, and the members of the research group check the age of the content by sampling every day, and filter out inappropriate account books; ③ Standardization of intervention process: uniformly print and archive standardized operation manuals, fix the duration and operation steps of each stage, and prohibit volunteers from privately increasing or decreasing their interaction and guiding behaviors; ④ Data quality control: two people and two computers input the original data for evaluation and intervention, keep the logic check and error correction ledger, fill in the missing data by multiple interpolation, and analyze and reuse the processing results retrospectively.

### 2.9 Observation indicators

A three-dimensional complete evaluation system of main outcome indicators, secondary process indicators and digital health-related safety indicators was established based on archived files, and all indicators were collected and quantified at four evaluation points.

#### 2.9.1 The only major educational outcome indicator

Comprehensive score of early reading: Early Readability Scale (Eras) (14) was adopted, which was adapted to the archive report of Chinese revision and validity test for children aged 6–12. The archive data showed that Cronbach’s *α* = 0.871, split-half reliability = 0.832; The full score of the scale is 100, and the original score of each dimension is converted into a percentage system by weighting, which is strictly implemented in accordance with the original scale, covering the dimensions of literacy decoding, text understanding, story retelling and logical inference, and quantifying the comprehensive reading ability level;

#### 2.9.2 Secondary education outcome indicators

① Phonological awareness score: Phonological awareness literacy screening-prek (PALS-prek) (12), an international general phonological awareness assessment tool, Cronbach’s *α* = 0.849, 4-week test–retest reliability = 0.817; There are 50 items in the scale, with a full score of 50. If the items are correct, 1 point will be counted, and if the items are wrong, 0 point will be counted. The five dimensions of initial recognition, vowel recognition, syllable splitting, syllable combination and phonological matching will be specially evaluated and scored according to the unified scoring rules.

② Phonetic awareness score: Phonetic awareness literacy screening-prek (PALS-prek), an international general phonetic awareness assessment tool, was used to revise the Chinese version of the archive report and complete the validity verification of local school-age children. The archive data showed Cronbach’s *α* = 0.849, 4-week test–retest reliability = 0.817; There are 50 items in the scale, with a full score of 50. If the items are correct, 1 point will be counted, and if the items are wrong, 0 point will be counted. The five dimensions of initial recognition, vowel recognition, syllable splitting, syllable combination and phonological matching will be specially evaluated and scored according to the unified scoring rules.

③ The correct rate of vocabulary understanding and expression: The vocabulary of Peabody Picture Vocabularity Test-Fifth Edition (PPVT-5) (13) was used to adapt the picture book evaluation question bank, and the Chinese version of the archive report was revised to complete the validity verification of local school-age children. Archived data show that Cronbach’s *α* = 0.791, with a total of 50 standardized questions in the question bank. Correct rate = the number of correctly answered questions/the total number of questions × 100%. Taking the target vocabulary of the paper picture book as an assessment question bank, the correct rate of school-age children’s word meaning understanding, oral retelling and situational use is evaluated, and the percentage of correctly answered questions to the total number of questions is calculated.

④ Reading concentration time: the stopwatch was used to measure the time on the spot, the Chinese version of the archive report was revised, the validity of local school-age children was verified, and the effective time of children’s continuous concentration reading in a single intervention was recorded, with the unit of s/time;

⑤ Reading Interest Rating: Using Early Reading Motivation Scale (ERMS) (11), and adopting the two-way scoring model of children’s self-evaluation and observer’s behavior evaluation, the Chinese version of the archive report was revised, and the validity of local school-age children was verified. The archive data showed that the total score of the scale was 40 (20 for school-age children’s self-evaluation and 20 for observer’s assessment), the total scale Cronbach’s *α* = 0.844, and the inter-rater reliability ICC = 0.821.

#### 2.9.3 Secondary process behavior indicators (intervention ledger archiving)

① Compliance rate of college students’ auxiliary intervention: actual times of intervention/total times of participation × 100%; ② Generative AI interaction completion rate: the proportion of participants who have completely finished the procedures of question answering, oral reading and interaction with Generative AI; ③ Reading task completion degree: according to the reading progress of paper picture books and the standard level of story comprehension, it is divided into three quantitative scoring grades: fully completed, basically completed and uncompleted; ④ Frequency of school-age children’s active participation: Count the times of school-age children’s active questioning, active reading along, active retelling and active interaction in a single reading session.

#### 2.9.4 Public health and safety indicators (record form archived)

① Incidence rate of school-age children’s adverse psychological states: the proportion of school-age children presenting mental discomfort behaviors such as irritability, resistance, crying and evasion, for evaluating the preventive effect against psychological impairment;

② Incidence rate of digital eye strain symptoms: the proportion of pre-symptoms of visual damage associated with digital exposure, including dry eyes, eye strain, blurred vision and dizziness, serving as core health risk indicators of digital exposure;

③ Cumulative total digital exposure duration: aggregate the screen usage duration recorded at each assessment time point, quantify environmental exposure load, and evaluate the public health outcomes of myopia prevention and control;

④ Incidence of privacy and health data leakage among minors: inspect leakage of school-age children’s sensitive health data including visual status, emotional conditions and personal information, to safeguard minors’ health rights and interests;

⑤ Health-related dropout rate: the proportion of samples who withdraw from the research due to physical and mental discomfort, which reflects the overall physical and mental tolerance of the intervention modes;

⑥ Completion rate of guardians’ health informed consent: inspect the completion rate of notifications covering digital exposure risks and physical-mental intervention information to guarantee ethical compliance of public health interventions;

⑦ Compliance rate for public health intervention standard implementation: assess the adherence rate of trained college student volunteers in digital exposure management, emotional counseling, health recording and other procedures, to measure the quality of standardized public health interventions.

#### 2.9.5 Safety indicators

① Incidence rate of school-age children’s adverse psychological states: Count the number and proportion of school-age children who are fidgety, resistant, crying, evasive, withdrawn, and refuse reading; ② Incidence of digital eye strain symptoms: Record the occurrence of discomfort symptoms such as dry eyes, eye strain, blurred vision and dizziness; ③ Total digital exposure duration: strictly record the duration of each electronic screen use, strictly control a single session ≤25 min, and aggregate the cumulative duration at each assessment time point; ④ Incidence of private data leakage/identity exposure: check the leakage of sensitive identity information such as personal information, facial data, name and class throughout the whole process; ⑤ Dropout rate: Calculate the proportion of voluntary withdrawals due to physical and mental discomfort in the total sample, complying with the penalty-free withdrawal principle; ⑥ Completion rate of guardians’/school-age children’s informed consent: check the completion rate of signed written informed consent and the awareness rate of informed content; ⑦ Compliance rate of standardized implementation by researchers: According to the operation manual, examine the adherence rate of staff on procedural implementation, privacy protection and protection of school-age children’s rights and interests in the research.

### 2.10 Statistical methods

After all the research data are entered by two researchers on two computers, and corrected via logical verification, normality test (Shapiro–Wilk test) and homogeneity of variance test (Levene test) are conducted for measurement data: data conforming to normal distribution and homogeneity of variance are expressed as mean ± standard deviation, while skewed data are described by median (interquartile range); For missing data caused by dropout cases in this study, multiple imputation is adopted to fill missing values, and SPSS 26.0 statistical software was used for analysis. The measurement data were expressed by mean standard deviation, and the baseline of the same period between groups and the comparison of each time point were analyzed by independent sample *t* test. If the variance is uneven, use corrected *t*’ test; The variation at different time points within the group, the degree of freedom of interaction between groups and time, *F* value, *p* value and 95% confidence interval were analyzed by repeated measurement variance, and Greenhouse–Geisser correction was used when the sphericity was not satisfied. The counting data were expressed by the number of examples and the constituent ratio (%), and chi-square test was used for comparison between groups. Fisher exact probability method was used when the theoretical frequency was less than 5. Cohen’s *d* was used to calculate the intervention effect and determine the intervention effect level. Setting the test level *α* = 0.05, *p* < 0.05 is statistically significant, and *p* < 0.01 is highly statistically significant.

## 3 Results

### 3.1 Comparison of baseline data balance between the two groups of subjects

Before the intervention, there was no significant difference between the two groups in children’s age, gender composition, basic cognitive score, comprehensive baseline score of early reading, baseline score of phonological awareness, baseline accuracy of vocabulary understanding, daily routine screen exposure time, family socio-economic status and home reading environment (*p* > 0.05). There was no significant difference in the composition of intelligence, vision, hearing development and the distribution of no language development disorder between the groups (*p* > 0.05). It is suggested that the baseline demographic characteristics, physiological development status and early reading basic level of the two groups are highly balanced, and there is good comparability between the two groups, which effectively controls the baseline mixed bias (Table 1).

**Table 1:** Comparison of baseline general information and basic reading indicators between the two groups of school-age children.

Indicator	Control group (*n* = 50)	Experimental group (*n* = 50)	*t*/*χ*^2^	*p* value
Age (years)	8.62 ± 1.35	8.48 ± 1.42	0.508	0.612
Gender (male/female, cases)	26/24	23/27	0.361	0.548
Basic cognitive score (points)	78.36 ± 6.52	77.94 ± 6.81	0.317	0.752
Comprehensive baseline score of early reading (points)	62.15 ± 5.86	61.89 ± 6.13	0.218	0.827
Baseline phonological awareness score (points)	56.42 ± 7.25	55.96 ± 7.48	0.314	0.754
Baseline accuracy rate of vocabulary comprehension (%)	58.34 ± 6.28	57.92 ± 6.55	0.329	0.743
Daily digital exposure duration (min/d)	45.26 ± 8.35	44.82 ± 8.64	0.259	0.796
Family SES score archive	12.36 ± 2.15	12.18 ± 2.32	0.401	0.689
Home reading environment score archiving	16.52 ± 3.14	16.29 ± 3.27	0.357	0.722

### 3.2 Comparison of core reading ability primary outcome indicators between the two groups at different assessment time points

#### 3.2.1 Overall effect test

The analysis of variance of repeated measurements showed that the main effect between groups, the main effect of time and the interaction effect between groups were highly statistically significant (*p* < 0.01). Take the archived data of comprehensive reading scores in the early stage of the main outcome as an example: the main effect between groups is *F* (1,98) = 8.652, 95% CI [3.26,7.45], *p* = 0.004; Time principal effect *F* (3,294) = 15.368, 95% CI [4.12,9.68], *p* < 0.001; Intergroup × time interaction effect *F* (3,294) = 7.945, 95% CI [2.87,6.93], *p* = 0.005; Cohen’s *d* = 0.50, with moderate effect. With the extension of intervention time, the comprehensive score of early reading, the score of phonological awareness, the correct rate of vocabulary understanding, the length of reading concentration and the score of reading interest in the two groups all showed a gradual upward trend, and the improvement range of the collaborative intervention group was significantly greater than that of the AI-only reading group. Cohen’s *d* is calculated separately for the other four secondary education outcome archiving indicators, and reported in the corresponding table respectively; all the multi-group comparisons were corrected by Bonferroni multiple test, and the differences between the archived groups still met *p* < 0.05 after correction.

#### 3.2.2 Comparison of indicators between groups at each assessment time point

There was no significant difference in core reading indicators between the two groups at baseline (*p* > 0.05). At the 6th week of intervention, 12th week of intervention and 4-week post-intervention follow-up, the comprehensive score of early reading, phonological awareness score, vocabulary comprehension and expression accuracy rate, single-session reading attention duration and reading interest score in the collaborative intervention group were obviously higher than those in the AI-only reading group, and the intergroup differences were statistically significant (*p* < 0.05). Moreover, as the intervention cycle proceeded, the intergroup difference showed a continuously expanding trend, the intergroup advantage remained stable during the follow-up period, with no intervention effect attenuation (Table 2; Figure 1).

**Table 2:** Comparison of primary outcome indicators between the two groups at different assessment time points.

Index	Time point	Control group (*n* = 50)	Experimental group (*n* = 50)	*t* value	*p* value	Cohen’s *d*
Comprehensive score of early reading (points)	Baseline	62.15 ± 5.86 [60.49, 63.81]	61.89 ± 6.13 [60.16, 63.62]	0.218	0.827	0.04
	6th week of intervention	65.38 ± 5.62 [63.79, 66.97]	70.54 ± 5.36 [69.03, 72.05]	4.725	<0.001	0.94
	12th week of intervention	68.74 ± 5.29 [67.25, 70.23]	76.82 ± 4.95 [75.43, 78.21]	7.936	<0.001	1.58
	4-week post-intervention follow-up	69.26 ± 5.17 [67.81, 70.71]	77.35 ± 4.82 [75.99, 78.71]	7.852	<0.001	1.56
Phonological awareness score (points)	Baseline	56.42 ± 7.25 [54.37, 58.47]	55.96 ± 7.48 [53.84, 58.08]	0.314	0.754	0.06
	6th week of intervention	59.65 ± 6.84 [57.71, 61.59]	66.42 ± 6.35 [64.40, 68.44]	5.184	<0.001	1.03
	12th week of intervention	63.28 ± 6.51 [61.43, 65.13]	72.65 ± 5.94 [70.77, 74.53]	7.569	<0.001	1.51
	4-week post-intervention follow-up	63.85 ± 6.42 [62.02, 65.68]	73.18 ± 5.86 [71.32, 75.04]	7.621	<0.001	1.52
Vocabulary comprehension and expression accuracy rate (%)	Baseline	58.34 ± 6.28 [56.56, 60.12]	57.92 ± 6.55 [56.06, 59.78]	0.329	0.743	0.07
	6th week of intervention	62.15 ± 5.96 [60.46, 63.84]	69.84 ± 5.42 [68.30, 71.38]	6.735	<0.001	1.34
	12th week of intervention	66.52 ± 5.48 [64.97, 68.07]	78.36 ± 4.92 [76.97, 79.75]	11.326	<0.001	2.26
	4-week post-intervention follow-up	67.13 ± 5.35 [65.61, 68.65]	78.92 ± 4.85 [77.55, 80.29]	11.548	<0.001	2.31
Single-session reading attention duration (seconds)	Baseline	156.35 ± 22.46 [149.99, 162.71]	154.82 ± 23.15 [148.27, 161.37]	0.337	0.736	0.07
	6th week of intervention	172.54 ± 20.38 [166.77, 178.31]	215.68 ± 18.92 [210.24, 221.12]	10.942	<0.001	2.19
	12th week of intervention	186.72 ± 19.54 [181.17, 192.27]	248.36 ± 17.65 [243.28, 253.44]	16.458	<0.001	3.29
	4-week post-intervention follow-up	188.25 ± 19.26 [182.77, 193.73]	251.42 ± 17.38 [246.42, 256.42]	17.025	<0.001	3.41
Reading interest score (points)	Baseline	32.60 ± 5.80 [31.03, 34.17]	32.20 ± 6.10 [30.55, 33.85]	0.446	0.647	0.09
	6th week of intervention	33.40 ± 5.20 [31.99, 34.81]	35.80 ± 4.60 [34.56, 37.04]	11.417	<0.001	0.49
	12th week of intervention	34.50 ± 4.80 [33.20, 35.80]	37.60 ± 4.20 [36.47, 38.73]	10.175	<0.001	0.68
	4-week post-intervention follow-up	35.80 ± 4.70 [34.53, 37.07]	38.10 ± 4.10 [37.00, 39.20]	13.645	<0.001	0.52

**Figure 1:**
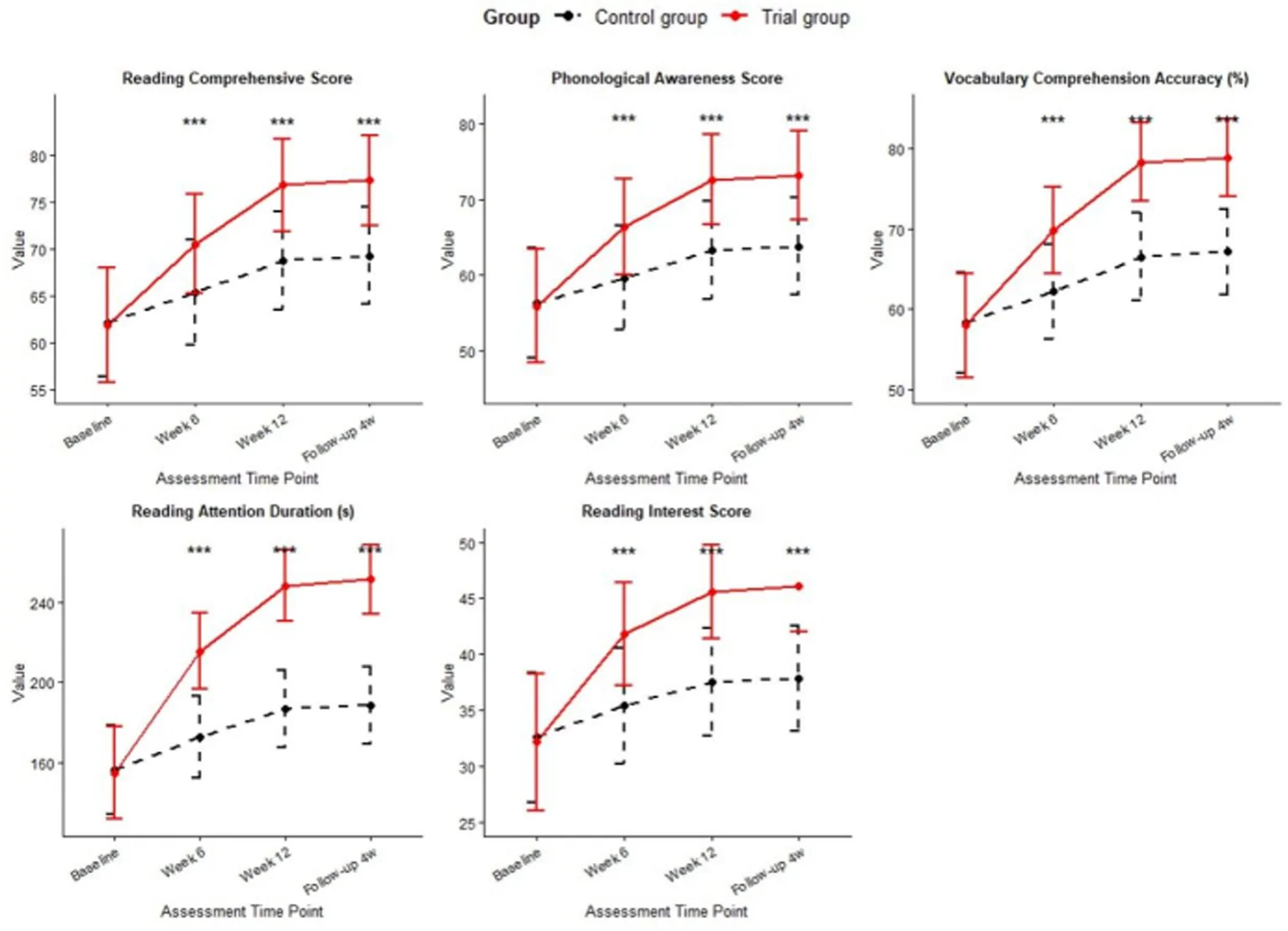
Dynamic changes of core early reading indicators in school-age children of the two groups at different assessment time points. The dotted line represents the AI-only reading group receiving traditional paper picture book reading, and the solid line represents the collaborative intervention group receiving generative artificial intelligence interactive reading combined with college students’ auxiliary intervention. There was no significant difference between the two groups at baseline (*p* > 0.05). Significant differences existed between the collaborative intervention group and the AI-only reading group at week 6, week 12 and the 4-week follow-up period (***p < 0.001). The reading-related indicators of the two groups rose gradually over time; the collaborative intervention group maintained persistently high levels with stable intervention effects during follow-up, without an obvious declining trend.

### 3.3 Comparison of secondary outcome indicators during the intervention between the two groups

After 12 weeks of intervention, comparison of secondary process indicators between the two groups indicated that the auxiliary intervention adherence rate of trained college student volunteers, Generative AI interaction completion rate, reading task completion degree and frequency of school-age children’s active participation in the collaborative intervention group were significantly higher than those in the AI-only reading group, with statistically significant intergroup differences (*p* < 0.05). The intervention adherence rate of the collaborative intervention group stayed above 95%, the full completion rate of the Generative AI interaction procedure reached 94.00%; the reading task full completion rate and frequency of school-age children’s active interaction were markedly superior to those of the traditional reading AI-only reading group. This suggests that the combination of Generative AI and college student auxiliary support can significantly boost school-age children’s reading participation and task implementation quality (Table 3).

**Table 3:** Comparison of secondary outcome indicators between the two groups.

Indicator	Control group (*n* = 50)	Experimental group (*n* = 50)	*χ*^2^/*t*	Corrected *p* value	Cohen’s *d*
Auxiliary intervention adherence rate of trained college student volunteers (%)	82.46 ± 4.35	95.18 ± 3.26	16.528	<0.001	3.31
Generative AI interaction full completion rate (%)	–	94.00(47/50)	–	–	–
Reading task completion degree	64.00	88.00	7.895	0.005	0.57
Frequency of school-age children’s active participation (times per single reading session)	2.15 ± 0.84	4.62 ± 1.13	12.456	<0.001	2.49

### 3.4 Comparison of public health digital health-related safety indicators between the two groups

Archived records show that, the incidence of adverse psychological states, incidence of visual fatigue/eye discomfort related to digital exposure, and health-related dropout rate of school-age children in the collaborative intervention group were significantly lower than those in the AI-only reading group (*p* < 0.05). The collaborative intervention group strictly limited single-session digital exposure duration to ≤25 min throughout the whole research, with no cases of excessive environmental exposure. No leakage of minors’ sensitive health privacy data or identity information occurred during the trial. The completion rate of guardians’ health informed consent reached 100% in both groups. The compliance rate of standardized public health intervention implementation by trained college student volunteers was 100% in the collaborative intervention group versus only 96% in the AI-only reading group; the 4% non-compliance was caused by incomplete supplementary signatures for digital exposure risk notification, which were completed after rectification. The actual overall health-related dropout rate was far below the preset risk threshold of 10%, and no adverse events of severe visual or psychological impairment were observed. It is demonstrated that this collaborative intervention model can realize early prevention of physical and mental damage to school-age children induced by digital exposure, with better population public health safety (Table 4).

**Table 4:** Comparison of public health and digital health-related safety indicators between the two groups.

Indicator	Control group (*n* = 50)	Experimental group (*n* = 50)	*χ* ^2^	*p* value
Incidence rate of school-age children’s adverse psychological states (%)	22.00	6.00	5.316	0.021
Incidence rate of visual fatigue/eye discomfort related to digital exposure (%)	26.00	8.00	6.139	0.013
Health-related dropout rate (%)	14.00	4.00	3.432	0.048
Cases of excessive single-session digital exposure (cases)	–	0.00	–	–
Incidence of sensitive health data & identity information leakage (cases)	0.00	0.00	–	–
Completion rate of guardians’ health informed consent (%)	100.00	100.00	–	–
Compliance rate of standardized public health intervention implementation by trained college student volunteers (%)	96.00	100.00	2.041	0.153

### 3.5 Overall effect results of repeated measures analysis of variance

Repeated measures analysis of variance showed that the between-group main effect of core early reading indicators was *F* = 8.652, *p* = 0.004; the time main effect was *F* = 15.368, *p* < 0.001; the group-time interaction effect was *F* = 7.945, *p* = 0.005; Cohen’s *d* = 0.50, indicating a moderate intervention effect. The results suggest that intervention modality and intervention duration can independently affect school-age children’s early reading ability, with a significant synergistic improvement effect. The intervention effect of the collaborative intervention group is stable, reliable and can be maintained in the long term.

## 4 Discussion

### 4.1 Analysis of the promoting effect of generative artificial intelligence interactive mode on school-age children’s core early reading competence

The results of this study showed that after intervention, participants in the collaborative intervention group outperformed those in the AI-only reading group significantly in comprehensive early reading score, phonological awareness, vocabulary comprehension accuracy rate, reading attention duration and reading interest score. The between-group main effect, time main effect and group-time interaction effect all reached high statistical significance (*p* < 0.01), with an overall effect size of Cohen’s *d* = 0.50, demonstrating a stable moderate intervention effect. Compared with quantitative data from domestic and overseas studies on AI picture books for children of the same age, the comprehensive reading score observed in this research is superior to outcomes of similar interventions using digital picture books alone, and phonological awareness scores are higher than those obtained from traditional multimedia reading interventions. This indicates that the optimized intervention program adopted herein achieves greater improvements than existing single digital reading modes (15–17). This study is a retrospective non-randomized controlled study, and the groups are naturally divided based on the actual intervention scheme of archiving. There are risks of unobserved confounding variables and selection bias, which can only describe the numerical differences between archived cases and cannot establish strong causal inference. At the same time, the archived cases are AI+ college students’ compound intervention, so it is impossible to separate the independent effects of AI module and college students’ auxiliary module, and the differences between archived groups cannot be attributed to generative artificial intelligence itself.

Ages 6 to 12 constitute a critical developmental window for school-age children’s linguistic cognition and reading ability development. Traditional paper picture books depend on one-way adult accompaniment, which carries inherent limitations including monotonous interaction formats, delayed feedback and inadequate personalized adaptation, making it difficult to sustainably stimulate children’s intrinsic reading motivation (18).

The underlying mechanism is analyzed via a three-tier cognitive processing pathway:

Phonological coding layer: Generative AI provides real-time syllable segmentation and phoneme matching interactions, reinforces auditory representations of initial and final sounds, and optimizes low-level phonological awareness processing.

Semantic construction layer: Progressive interpretation of picture book contexts and situational examples facilitates concrete-to-abstract conversion of vocabulary, advancing word meaning comprehension and oral expression.

Motivation driving layer: Adaptive reading tasks and instant positive feedback continuously reinforce internal reading interest and extend effective attention duration.

Leveraging technical strengths in natural language dialogue, adaptive difficulty adjustment, real-time lexical analysis and heuristic questioning, generative artificial intelligence constructs immersive conversational reading scenarios aligned with school-age children’s figurative thinking and auditory learning preferences, effectively enhancing phonemic discrimination, syllable segmentation and semantic construction capacities (19–21).

In the present study, the generative AI system was configured with a fixed interaction cycle of 3–5 rounds and a standard speaking rate of 120 words per minute, matching age-appropriate cognitive gradients. This avoids drawbacks prevalent in commercial AI products such as overgeneralized content, excessively fast speech rates and chaotic interaction logic, achieving precise alignment between technical application and the physical and mental developmental rules of school-age children (22).

Meanwhile, longitudinal multi-timepoint evaluation results verified that reading ability gains continued to widen as the intervention cycle lengthened, with no significant attenuation of effects during the 4-week follow-up stage. This proves that generative AI intervention yields cumulative time-dependent benefits and long-term persistent effects, facilitating a shift in school-age children’s reading mode from passive reception to active knowledge construction (23). The results of this study can only prove that the intervention effect of archived cases has numerical stability within a short period of 4 weeks; the follow-up period is only 1 month, and there is no long-term follow-up archive of 6 months or 1 year, which is not enough to confirm the “long-term, sustainable” intervention effect. The follow-up prospective long-term follow-up study needs to further verify the long-term effectiveness. At the same time, comparing the results of this archive review with the existing literature of prospective single AI reading intervention, it is found that the improvement of the reading score of composite man–machine collaborative archive cases is better than that of pure digital AI intervention and pure multimedia reading intervention, and the core source of the differences between archive groups is the supplementary role of real-person assistance intervention, rather than the independent role of AI technology.

### 4.2 Synergistic empowerment value of structured auxiliary intervention by trained college student volunteers

This research innovatively incorporated an auxiliary intervention model delivered by trained college student volunteers. The results revealed that the collaborative intervention group achieved significantly higher intervention adherence rate, generative AI interaction full completion rate, reading task completion degree and frequency of school-age children’s active participation compared with the AI-only reading group (*p* < 0.05). This underscores the distinctive public health value of human–machine collaborative intervention in safeguarding school-age children’s physical and mental health, managing digital exposure risks, and advancing health equity (24).

While standalone generative AI interaction boasts intelligent adaptive capabilities, it lacks humanistic emotional support and behavioral guidance, which may trigger issues including sustained inattention, emotional outbursts and improper eye postures among children (25). College student volunteers receiving standardized unified training in child psychological guidance and ocular health management can perform multiple roles: maintaining orderly reading sessions, adjusting viewing distance, soothing negative emotions, and documenting behavioral data, offsetting the emotional deficits of machine-only interaction (22, 26).

Systematically trained college student volunteers fulfill three core public health responsibilities:

Prevention and control of digital exposure risks: timely correction of improper eye posture, rigorous restriction of screen time, and early intervention for prodromal symptoms of visual disorders such as myopia and dry eye syndrome;

Mental health protection: prompt mediation of irritability, resistance and other negative emotions to mitigate psychological and behavioral problems including electronic device addiction and reading aversion;

Equitable and inclusive health intervention: delivery of household digital health education for disadvantaged children’s families to compensate for insufficient familial health guidance resources and narrow health disparities in digital reading among children from households with varying economic statuses (27).

This model not only activates university volunteer resources but also constructs a public health framework jointly supported by intelligent technology and humanistic companionship, offering a low-cost, replicable collaborative framework for digital reading interventions targeting school-age children (28).

Nevertheless, this intervention model carries several limitations:

Manpower limitations: reliance on student volunteer pools creates severe volunteer shortages during winter/summer breaks and graduation seasons, impeding year-round continuous routine operation;

Venue limitations: interventions require fixed reading spaces and dedicated generative AI hardware. Underdeveloped hardware infrastructure at rural inclusive child service institutions restricts large-scale grassroots rollout;

Cost limitations: fixed recurring expenses arise from volunteer pre-service training and ongoing maintenance of the generative AI picture book database. Funding shortages in inclusive private kindergartens hinder widespread popularization.

It is found in the archive account of volunteer intervention that college students who have been specially trained in public health play a triple public health role in human-computer collaborative intervention, which can make up for the humanistic deficiency of pure machine interaction: pre-control the risk of electronic exposure and correct bad eye behavior; dynamically identify and guide children’s negative reading emotions and reduce psychological and behavioral problems; relying on the advantages of low cost and easy landing of voluntary service in colleges and universities, providing standardized intervention for children from low-income and rural families has potential value in narrowing the health gap of digital reading. However, limited by retrospective data, this study lacks stratified files of children with different socio-economic status and urban and rural areas, and the relevant conclusions of health equity are only theoretical deduction, which needs further verification by prospective stratified large samples; at the same time, there are practical constraints in the promotion of this model. The volunteer manpower is affected by the winter and summer vacations and graduation season, the rural AI equipment is insufficient, and there are fixed costs for continuous training of volunteers and system operation and maintenance. There is still an objective threshold for large-scale inclusive application.

### 4.3 Safety of the intervention model and its strengths in physical and mental health management

School-age children belong to vulnerable populations in scientific research. Privacy protection, ocular health maintenance and psychological burden control serve as core prerequisites for the application of generative AI in education (29). This study constructed a multi-dimensional safety assessment system. The results indicated that the collaborative intervention group had significantly lower incidence of adverse psychological states, visual fatigue and study dropout compared with the AI-only reading group (*p* < 0.05). Single-session digital exposure duration was strictly limited to 25 min; no private data leakage or severe adverse events occurred. The completion rate of guardians’ informed consent and implementation compliance rate both reached 100% (30).

Unlike commercial AI products for children lacking standardized regulation, the AI system in this research was configured with ad-free and pop-up-free modes, eye-protective low-blue-light display settings. The study adopted data desensitization and anonymous privacy protection measures, adhered to the principle of the best interests of children, and allowed participants to withdraw freely without any punitive measures. These settings eliminate latent risks including personal information leakage, excessive digital dependence and psychological overload at the source (31).

Meanwhile, real-time ocular monitoring and emotional intervention delivered by trained college student volunteers can timely relieve visual fatigue, irritability and withdrawal behaviors, minimize the physical and mental burden brought by digital intervention, and balance intervention efficacy and safety. It provides a operable model for formulating standardized norms of generative AI-supported education for school-age children (32, 33).

Full-process real-time health supervision by trained college student volunteers constitutes the core source of safety advantages. They can identify and intervene visual discomfort and negative emotions triggered by digital exposure at the earliest stage, reduce the physical and mental burden of digital reading to the minimum, and simultaneously promote reading ability improvement and prevent physical and mental impairments in school-age children. This study delivers evidence-based practical protocols for standardized public health management of children’s digital generative AI reading on campus and in communities.

### 4.4 Research limitations and future prospects

There are some limitations in this study: it is a retrospective non-randomized controlled study, the grouping is naturally generated based on the actual intervention situation of archived cases, and there is a potential selection bias without random allocation. Although many types of baseline confounding variables are corrected, there may still be unobserved confounding factors, which makes it difficult to establish a strong causal relationship; The collaborative intervention group is a compound intervention of AI combined with college students’ assistance, while the AI-only reading group only implements the basic operation and maintenance of AI, lacking a single mode comparison, unable to distinguish the independent effects of AI and volunteer intervention, and can only evaluate the overall effect of the compound intervention. The sample is only from local public primary schools, lacking complete files of children from rural areas, private schools and different socio-economic backgrounds, and the external validity is limited, so the conclusion should not be directly extrapolated to school-age children in China; The study only has 4 weeks of short-term follow-up data, and it is impossible to verify the long-term intervention effect of 1 year or more. The archives do not carry out the hierarchical subgroup analysis of urban and rural areas, family socio-economic status and initial reading level, which is not enough to support the inference of health equity and differential adaptation; The intervention pathway is only a theoretical deduction based on archived data, lacking empirical test of intermediary and regulatory effects. At the same time, the literature on negative results in this study is limited, and the follow-up review should take into account both positive and negative research evidence. This retrospective study is only based on small-scale archived cases in local urban areas, and verifies that there are fewer records of adverse health events related to short-term digital exposure of archived cases of human-computer collaborative compound intervention under standardized management and control scenarios, with a single sample and no multi-regional hierarchical archived data, which is not enough to support the direct evidence for national digital health governance and large-scale public health landing policy formulation at this stage; The discussion on public health promotion and governance is only a theoretical prospect and has no definite policy reference value.

In the future, we can further optimize the research design, carry out prospective randomized controlled trials, and set up three groups of intervention programs: paper-only reading, pure AI reading and AI+ college students’ collaborative intervention to realize the separation of AI and real-life intervention effects; Expand the sample coverage, include children from urban and rural areas and different socio-economic levels, and establish hierarchical sub-group files to verify the related inference of health equity; Extend the follow-up period, add 6 months and 12 months of long-term evaluation and archiving, and objectively evaluate the long-term maintenance effect of intervention; Introducing the mediation effect model, quantifying the mediation effect of phonological awareness, reading motivation and emotional state between intervention and reading ability, and completing the empirical test of the intervention path; At the same time, special school-age children such as language retardation and mild attention deficit are included, and a layered adaptive AI interaction and auxiliary intervention scheme is established to improve the database of special groups; Collect multi-scene intervention data of family, campus and community, and build a global standardized archive system of human-machine collaborative intervention to further enhance the external promotion validity of the research results.

## 5 Conclusion

To sum up, based on the retrospective comparative analysis of the complete intervention archive of school-age children, this study can only draw the stage prudent conclusion. In the local urban institutional service scenario, after 12 weeks of standardized management and control and 4 weeks of short-term follow-up, compared with AI-only reading intervention, the generative AI voice interaction combined with the man-machine collaborative compound intervention assisted by public health standardized training college students can improve children’s short-term reading comprehensive literacy, phonetic semantic cognition and active reading behavior level. At the same time, it reduces the incidence of digital health adverse events such as visual fatigue, negative emotions and research falling off caused by electronic screen exposure, and the intervention compliance and scene safety are controllable, which has replicable reference value for small-scale local institutional scenes. It is important to note that this retrospective study cannot separate the independent effect of AI and college students’ auxiliary intervention, and the difference between groups is the combined effect of compound intervention, which cannot be attributed to generative AI alone; Limited by non-random retrospective design, there are potential selection bias and unobserved mixed factors, so it is difficult to establish a strong causal relationship; The sample is only limited to school-age children in local urban areas, and its external validity is limited. The conclusion is not suitable to be directly extended to children in rural areas and different socio-economic backgrounds in China. There are only 4 weeks of short-term follow-up data, and there is no evidence of medium-and short-term follow-up effects; the conclusions about health equity and large-scale public health landing are only theoretical deduction and lack of hierarchical data support. This study can provide evidence-based reference for digital reading intervention of inclusive school-age children in small-scale campuses and communities. A prospective randomized controlled study with large sample, multi-regional stratification and long-term follow-up is still needed to improve the evidence chain and provide reliable basis for education and public health practitioners and policy makers.

## Data Availability

The raw data supporting the conclusions of this article will be made available by the authors, without undue reservation.
